# Patient-Specific Induced Pluripotent Stem Cells for SOD1-Associated Amyotrophic Lateral Sclerosis Pathogenesis Studies

**Published:** 2014

**Authors:** I. V. Chestkov, E. A. Vasilieva, S. N. Illarioshkin, M. A. Lagarkova, S. L. Kiselev

**Affiliations:** Vavilov Institute of General Genetics RAS, Gubkina Str., 3, Moscow, Russia, 119991; Research Center of Neurology RAMS, Volokolamskoye shosse, 80, Moscow, Russia, 125367

**Keywords:** induced pluripotent stem cells, amyotrophic lateral sclerosis, differentiation, motor neurons

## Abstract

The genetic reprogramming technology allows one to generate pluripotent stem cells for individual patients. These cells, called induced pluripotent stem cells (iPSCs), can be an unlimited source of specialized cell types for the body. Thus, autologous somatic cell replacement therapy becomes possible, as well as the generation of *in vitro *cell models for studying the mechanisms of disease pathogenesis and drug discovery. Amyotrophic lateral sclerosis (ALS) is an incurable neurodegenerative disorder that leads to a loss of upper and lower motor neurons. About 10% of cases are genetically inherited, and the most common familial form of ALS is associated with mutations in the *SOD1 *gene. We used the reprogramming technology to generate induced pluripotent stem cells with patients with familial ALS. Patient-specific iPS cells were obtained by both integration and transgene-free delivery methods of reprogramming transcription factors. These iPS cells have the properties of pluripotent cells and are capable of direct differentiation into motor neurons.

## INTRODUCTION

Amyotrophic lateral sclerosis (ALS, also known as the motor neuron disease (MND), Charcot disease or Lou Gehrig’s disease) is an adult-onset progressive neurodegenerative disease that involves selective death of motor neurons in the brain and spinal cord. Gradual muscle de-innervation is observed during disease progression, and the death usually occurs from respiratory muscle failure. There is no effective early diagnosis of ALS and, therefore, patients live no more than 5 years after the manifestation of the first symptoms. Most cases of ALS (about 90%) are not genetically inherited and are known as sporadic ALS. The most common case of familial ALS (ALS1, 20% of all ALS patients) is associated with autosomal dominant mutations in the Cu/Zn superoxide dismutase (*SOD1*) gene. Over 170 mutations in the *SOD1 *gene have been characterized in ALS patients (ALSoD database, June 28, 2013).

However, the relationship between the genetic defect and the loss of motor neurons has not yet been established. Generation of transgenic mice with the mutant human *SOD1 *allele made it possible to reproduce the basic symptoms of neurodegeneration in ALS and test several hypotheses on the basis of disease pathogenesis. It was shown that the toxic effect of *SOD1 *on motor neurons is not associated with impairment or loss of its enzyme activity. Similar symptoms of ALS, including the loss of close synaptic contacts between motor neurons [1], disruption of the mitochondrial function [2], and activation of glial cells [3], have been confirmed in *SOD1*-trangenic mouse models expressing both dismutase-active (hSOD1^G37R^ [4], hSOD1^G93A^ [5]), and non-active (hSOD1^G85^R [6], hSOD1^G127X^ [7]) mutants. Moreover, homozygous *SOD1*–deficient mice showed no symptoms of neurodegenerative disorders [8].

The catalytic activity of SOD1 depends on the presence of a copper ion in the active site of the enzyme. In its free state, this metal ion exhibits extreme reactivity and toxicity; therefore, inefficient delivery of copper into the active site of SOD1 or its binding violation due to conformational changes in the enzyme (due to mutations) may prove the reason behind intracellular disorders and the death of motor neurons. However, removal of the copper chaperone of SOD1 (CC S) [9] or introduction of mutations in the copper-binding site of the enzyme [10] failed to reduce the selective toxicity to motor neurons in mice.

Nevertheless, conformational changes and the toxicity of mutant SOD1 are currently considered as the main cause of ALS. The loss of copper and zinc ions in the active protein site or the disruption of intramolecular disulfide bonds, leads to dissociation of native SOD1 homodimer into monomers and subsequent formation of protein aggregates, the presence of which is a characteristic of typical ALS [11]. Furthermore, the presence of SOD1-protein aggregates in a sporadic ALS patient’s motor neurons suggests that aberrant oligomerization of SOD1 is a common feature of ALS, regardless of genotype [12]. The conformational theory is supported by the fact that there are forms of the disease with different manifestation and progression rates that are dependent on the type of mutation in the *SOD1 *gene.

It should be noted that the death of motor neurons in ALS may be not an autonomous cell process, since the expression of *SOD1 *mutants selectively in motor neurons does not lead to neurodegeneration in transgenic mice [13], while a toxic effect has been shown for astrocytes [14] and microglial cells [3].

However, the results obtained in transgenic animalscannot always be directly transferred to humans. Systems overexpressing the mutant *SOD1 *gene may fail to reproduce the molecular basis of the disease progression. Besides, the study of human ALS is limited by the inaccessibility of affected tissues. The cell reprogramming technology allows the reprogramming of any somatic patient’s cells, for instance, skin fibroblasts [15] endothelial cells [16, 17], to the pluripotent state. These induced pluripotent stem cells (iPSCs) display all the characteristics of embryonic stem cells (ESCs), including the unlimited proliferative potential and the ability to differentiate into all cell types of the body.

In this article, we obtained iPS cells from patients with familial forms of *SOD1*-mediated ALS by reprogramming primary skin fibroblasts using ectopic expression of four transcription factors Oct3/4, Sox2, c-Myc, and Klf4. These iPSC lines have been characterized, and the protocol of their differentiation into motor neurons has been developed.

## EXPERIMENTAL

**Primary Fibroblast Culture**

Skin biopsies were plated under glass coverslips in Petri dishes (Greiner Bio-One) coated with 0.1% gelatin (Sigma) and cultured in Dulbecco’s modified Eagle medium (DMEM) (PanEco) supplemented with 10% fetal bovine serum (FBS) (Hyclone), 1% nonessential amino acids (Invitrogen), 2 mM L-glutamine (Invitrogen), 50 units/mL penicillin and 50 µg/mL streptomycin (PanEco), and 4 ng/ml hrbFGF (PeproTech) (fibroblast medium). At approximately day 14^th^ the confluent cell monolayer was formed, and cells were passaged with 0.25% trypsin (Hyclone). For cryoconservation, fibroblast cells were frozen in DMEM with 20% FBS and 10% dimethyl sulfoxide (DMSO).

**Cell Culture**

Primary dermal fibroblasts were cultured as described above. Phoenix retroviral packaging cells were cultured in DMEM with 5 % heat-inactivated (56°C) fetal bovine serum, 2 mM L-glutamine, 50 units/mL penicillin and 50 µg/mL streptomycin in Petri dishes coated with 0.1% gelatin. iPS cell lines were cultured in mTeSR1 medium (STE MCE LL Technologies) in Petri dishes coated with Matrigel (BD). All cell lines were incubated at 5% CO_2_ and 37°C.

**Recombinant Lentiviruses Production**

For the production of recombinant lentiviruses, a Phoenix packaging cell line was transfected with the lentiviral vectors encoding the reprogramming factors LeGOhOct3/ 4, LeGO-hSox2, LeGO-hKlf4, LeGO-hc-Myc as previously described [18].

**Transfection of Fibroblasts and iPS Cells Generation**

Fibroblasts were reprogrammed by the non-integrating method using a CytoTune-iPS Sendai Reprogramming Kit (Invitrogen) according to the manufacturer’s instructions. ;

To reprogram the cells by recombinant lentivirus particles, the fibroblasts at passage 1-2 were seeded at a density of 1 × 10^5^ per well of a 6-well plate (Greiner Bio- One) and incubated overnight in the fibroblast medium. The next day, the fibroblast medium was replaced with the fresh medium containing 8 mg/mL of polybrene (Sigma) and incubated for an hour. Then, the cells were incubated with virus-containing supernatants (MOI 5 for each lentivirus). The medium was changed every other day. Five days after transfection, the cells were passaged with 0.25% trypsin to mitomycin C-treated (10 µg/ml, Sigma) mouse embryonic fibroblasts (MEF) on 100-mm Petri dishes coated with 0.1 % gelatin. On the sixth day, the fibroblast medium was replaced with the human ESCs culture medium of the following composition: DMEM/F12 (1:1) (PanEco), 20% Knockout Serum Replacement (KO SR, Invitrogen), 1% nonessential amino acids, 1 mM L-glutamine, 50 units/ml penicillin, 50 µg/ml streptomycin, 0.1 mM beta-mercaptoethanol (Sigma), 4 ng/ml hrbFGF containing 1 µM BIX-01294, inhibitor of histone methyltransferase G9a (Sigma), and 1 µM valproic acid, histone deacetylase (HDAC) inhibitor (Sigma). The medium was changed every day. BIX-01294 and valproic acid were added to the medium for 7 days. Clones which were morphologically indistinguishable from ES cells were manually selected and cultivated in a mTeSR1 medium in Matrigel-covered cell culture dishes.

**Visualization of endogenous alkaline phosphatase activity**

The culture medium was aspirated, and the cells were washed twice with a buffer solution containing 100 mM Tris-HCl, 100 mM NaCl, 5 mM MgCl2, and 0.05% Tween-20, pH 9.5. Then, the buffer was aspirated and the working solution supplemented with 0.02% BCIP, 0.03% NBT in 0.1 M TBS (Tris-Buffered Saline), pH 9.5, was added. The cells were incubated for 30 min at room temperature, washed twice with the buffer solution, and positively stained clones were counted by light microscopy.

**Karyotyping and immunostaining**

Metaphase Chromosome Spread Preparations of iPSC lines and the immunostaining of cells were performed as previously described [17].

The following primary antibodies were used: mouse monoclonal antibody against SSEA-4 (1:100), TR A-1-60 (1:100), TR A-1-81 (1:50) (Cell Signaling Technology), HB9 (1:50, Developmental Hybridoma Bank or DSHB), βIII-tubulin, GFAP (1:500, Abcam), and vimentin (1:200, Daco); rabbit monoclonal antibody against Nanog (1:200), Oct4 (1:300), Sox2 (1:400) (Cell Signaling Technology), rabbit polyclonal antibody against ChAT, beta-tubulin, GFAP (1:500, Abcam), alpha-fetoprotein (1:400, Dako). The secondary antibodies included Alexa Fluor 555 goat anti-rabbit IgG (1:700, Invitrogen) and Alexa Fluor 488 goat anti-mouse IgG (1:500, Invitrogen).

The nuclei were counterstained with DAPI. The preparations were analyzed with an Axio Imager A1 epi-fluorescence microscope (Carl Zeiss). Pseudo color images of the micro-objects were obtained using the AxioVision software (Carl Zeiss).

**Formation of the embryoid bodies and their differentiation *in vitro***

Spontaneous differentiation of pluripotent cells was induced as previously described [18].

**Neuronal differentiation**

The differentiation protocol, with some modifications, was used [19] to prepare motor neurons *in vitro*. Embryoid bodies were prepared using an AggreWell 400 Plate (STE MCE LL Technologies) in DMEM/F12 medium supplemented with 5% KO SR, 1% nonessential amino acids, 2 mM L-glutamine, 50 units/ ml penicillin, 50 µg/ml streptomycin, 0.1 mM beta-mercaptoethanol, 200 ng/ml recombinant human Noggin protein (Biological Industries), and 2 µM SB431542 (Stemgent) for 12 days. Afterwards, embryoid bodies were cultured for a additional 10 days in Neurobasal Medium (Gibco) with 2 mM L-glutamine, B27 (Gibco), 1 µM retinoic acid, 200 ng/ml recombinant human Sonic hedgehog (PeproTech), 10 ng hrbFGF, and then transferred to Matrigel-coated Petri dishes with Neurobasal Medium containing 2 mM L-glutamine, 10 ng/ml BDNF, and 10 ng/ml GDNF (all from PeproTech) and cultured for another 14 days. Adherent cell colonies were treated with Accutase (Sigma) to dissociate them into single cells and cultured in Matrigel-coated Petri dishes for 5 days, after which the immunostaining was performed.

The directed differentiation of iPS cells into functional astrocytes was performed as described above [20].

## RESULTS AND DISCUSSION

**Genetic reprogramming of the skin fibroblasts of patients with familial ALS**

**Fig. 1:**
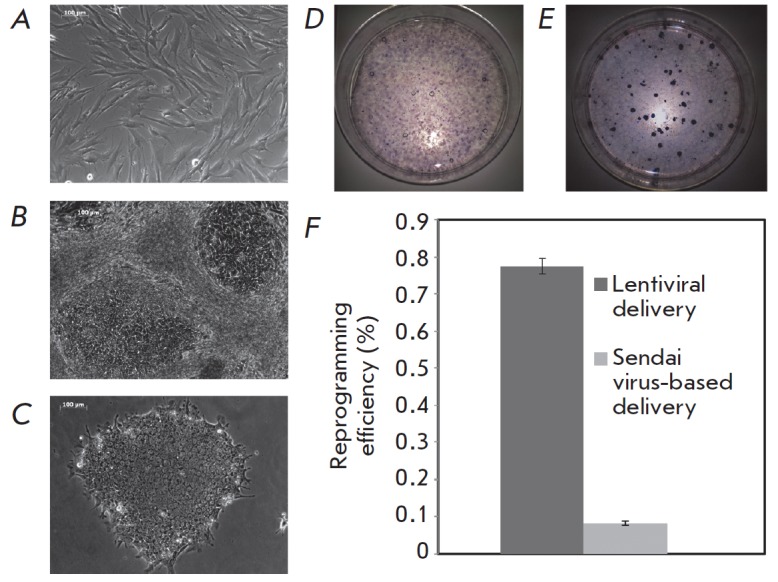
Generation of stable iPSC lines from the primary skin fibroblasts of patients with familial ALS. A – primary culture of skin fibroblasts; B – formation of iPS cell colonies after introduction of reprogramming genes (day 15); C – mechanically picked iPSC colony in feeder-free culture conditions; D, E – visualization of iPS colonies obtained with lentiviral (D) and Sendai virus-based systems (E) by alkaline phosphatase staining; F – comparison of the efficiency of two reprogramming systems

Skin biopsies of patients with ALS-characterized forms were provided by the Research Center of Neurology, RAMS. Homogeneous cultures of primary skin fibroblasts were obtained from these biopsy materials. The migration of fibroblasts occurred within two weeks prior to the formation of the first cell monolayer. No later than after 1–2 passages, the primary fibroblasts were transfected using lentiviral or recombinant Sendai virus-based delivery systems by four transcription factors: Oct3/4, Sox2, c-Myc, and Klf4. To increase efficiency of reprogramming, a methyltransferase inhibitor (BIX-01294) [21] and a histone deacetylase inhibitor (valproic acid) [22] were added to the culture medium. The induction of pluripotency in somatic cells is accompanied by a cascade of epigenetic events, including methylation of the gene promoters expressed in the differentiated cell types, hypomethylation of promoters and activation of pluripotency genes, as well as global chromatin changes and reactivation of a somatically silenced X chromosome [17]. Starting on day 11 after transduction, compact colonies, consisting of actively growing cells with an increased nucleus/cytoplasm ratio compared to the primary fibroblast culture (*Fig. 1 A, B*), were formed. Since these cell colonies were morphologically similar to ESCs, we held the mechanical selection of individual colonies to produce stable iPSC lines (*Fig*. *1C*). Staining for alkaline phosphatase (whose activity is elevated in pluripotent cells [23]) showed that the reprogramming efficiency increased 10-fold when the lentiviral gene delivery system was used (0.77 ± 0.025%), as compared with the recombinant Sendai virus delivery technique (0.083 ± 0.006%) (*Fig. 1 D, E, F*). Moreover, the stabilization of the pluripotent state using the Sendai virus-based method in the obtained transgene-free iPSC lines occurred gradually, over several passages, and it was accompanied by a high percentage of spontaneously differentiated cells (data not shown). Nevertheless, the use of the transgene- free delivery method allowed us to obtain genetically unmodified pluripotent stem cells for each patient. That is very important both for cell replacement therapy and for studying disease pathogenesis, since transgene integration into active chromatin sites may lead to changes in gene expression.

**Characteristics of patient-specific iPSC lines**

**Table:** Comparison of short tandem repeat (STR) profiles for the primary skin fibroblasts (PSF) of patients with familial ALS and isolated iPSC lines

STR- marker	PSF1	iPSC1.2	PSF2	iPSC2.2
AMEL	X Y	X Y	X X	X X
CSF1PO	10 14	10 14	10 12	10 12
D10S1248	13 14	13 14	14 15	14 15
D12S391	16 23	16 23	20 23	20 23
D13S317	11 12	11 12	11 11	11 11
D16S539	12 12	12 12	11 13	11 13
D18S51	15 15	15 15	13 14	13 14
D1S1656	13 17.3	13 17.3	12 16.3	12 16.3
D22S1045	15 16	15 16	15 16	15 16
D2S441	10 13	10 13	11 14	11 14
D3S1358	15 16	15 16	17 18	17 18
D5S818	11 11	11 11	12 13	12 13
D7S820	9 11	9 11	10 12	10 12
D8S1179	13 14	13 14	14 15	14 15
FGA	21 23	21 23	21 21	21 21
SE33	16 30.2	16 30.2	26.2 26.2	26.2 26.2
TH01	9 9.3	9 9.3	6 9.3	6 9.3
TPOX	9 11	9 11	8 8	8 8
vWA	17 18	17 18	17 18	17 18

**Fig. 2:**
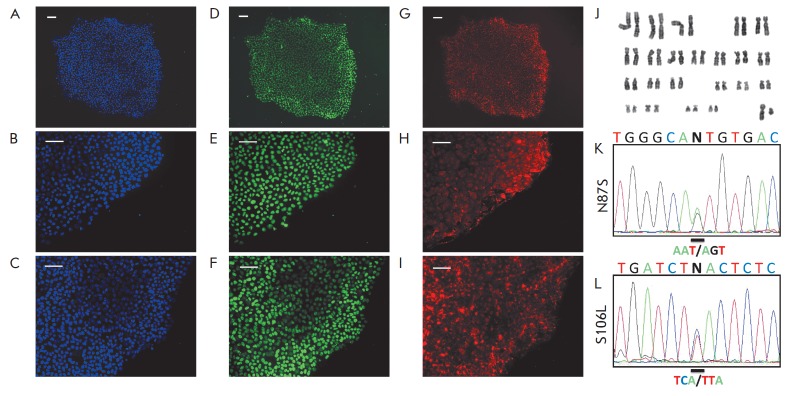
Characterization of patient-specific iPS lines. A–I – immunofluorescent analysis of the expression of pluripotency markers, including the transcription factors Oct3/4 (D), Sox2 (E), Nanog (F), and surface antigens SSEA4 (G), Tra-1-60 (H), Tra-1-81 (I). DAPI staining indicating the total cell count per field (A, B, C); J – karyotype of iPSC lines, GTG-banding; K, L – nucleotide sequences with mutations in the *SOD1 *gene

**Fig. 3:**
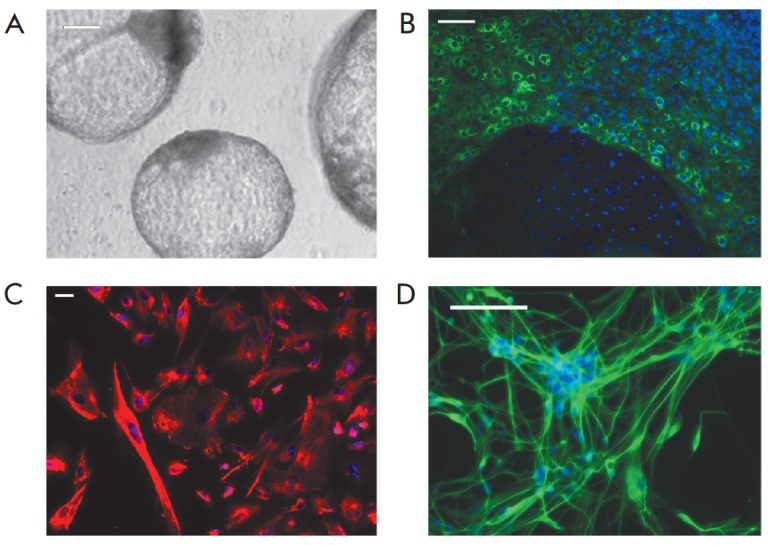
Spontaneous differentiation of iPS cells into three germ layers. A – representative image of embryoid bodies; B, C, D – immunocytochemical staining of α-fetoprotein (B), vimentin (C), βIII-tubulin (D). Cell nuclei stained with DAPI (blue)

According to immunocytochemistry results, iPSC lines expressed both ESC-specific surface antigens (SSEA- 4, TR A-1-60, TR A-1-81) and the nuclear transcription factors associated with pluripotency (Nanog, Oct4, Sox2) (*Fig. 2 A-I*). The GTG-banding of iPSC lines revealed no changes in the number and structure of chromosomes during reprogramming (*Fig. 2J*). In order to determine the ability of iPS cells to differentiate into all three germ layers, the analyzed lines were placed in the suspension culture, where they formed embryoid bodies. On day 14 after cultivation, embryoid bodies were transferred to gelatin-coated culture dishes for adhesion and following cell migration. The adherent cells showed various types of morphologies; immunocytochemistry revealed cells positive for βIII-tubulin (a marker of ectoderm), vimentin (mesoderm), and α-fetoprotein (AFP, endoderm) (*Fig. 3*).

To confirm the fact that isolated iPSCs were derived from the patient’s skin fibroblasts, we checked their short tandem repeat (STR ) profiles. We found that the patterns of 18 STR s (*Table*) were completely matched between isolated iPSCs and primary skin fibroblasts. Additionally, *SOD1 *gene mutations different for each patient were detected in iPS cell lines (*Fig. 2 K, L*).

Thus, according to cell morphology, pluripotency markers expression and the ability to differentiate into derivatives of three germ layers, the iPS cells derived from the fibroblasts of the patients with SOD1-associated ALS were pluripotent cells.

**Generation of motor neurons from the SOD1 mutant of iPS cells**

**Fig. 4:**
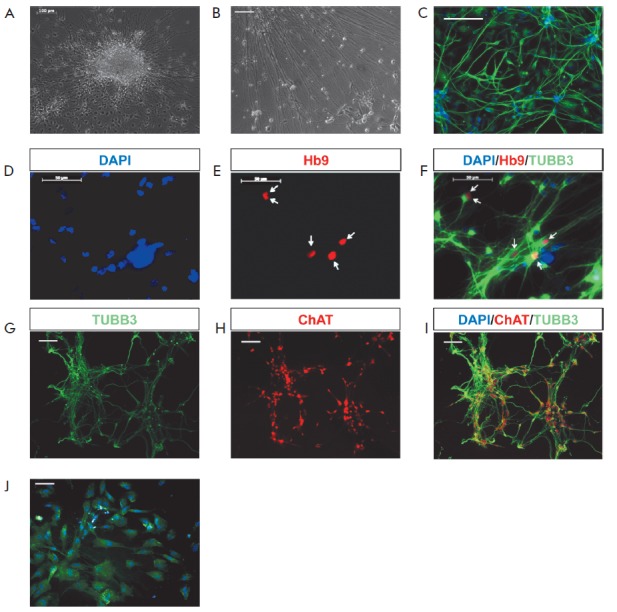
Neuronal differentiation of ALS patient-specific iPS cells. A, B – representative images of differentiated neuronlike cells; C – immunocytochemical staining of βIII-tubulin-positive cells; D–I – cells stained for the motor neuron markers Hb9 (E, F) and ChAT (H, I); J – detection of GFAP in iPSC-derived astrocytes. Cell nuclei stained with DAPI (blue)

As previously reported, the death of motor neurons in ALS patients might be a non-cell-autonomous process but depends on the cellular microenvironment [3, 14]. Therefore, our next aim was to develop protocols for the differentiation of iPSCs into motor neurons and astrocytes. Previously, it was shown *in vivo *that during development, motor neurons are forming by the exposure of rostral neural progenitors to two consecutive signals: retinoic acid (caudalization) and Sonic hedgehog (ventralization) [24]. These rostral neural progenitors were obtained from embryoid bodies suspension by inhibiting the Smad signaling pathway. The direct differentiation of iPSCs into motor neurons was performed 12 days later by adding retinoic acid (RA) and recombinant Sonic hedgehog (Shh) to the culture medium. After dissociation of neurospheres to single-cell suspension, neuronal precursors were transferred on Matrigel-coated dishes and cultivated as an adherent culture. These cells express the common neuronal marker βIII-tubulin, proving that the obtained cells are of neuronal origin (*Fig. 4 A-C*). In the last step, maturation of motor neurons occurred under the influence of neurotrophic growth factors, such as the brain-derived neurotrophic factor (BDNF) and glial cell line-derived neurotrophic factor (GDNF). Immunocytochemical staining of these cultures showed that βIII-tubulin-positive neurons co-expressed markers such as Hb9 (MNX1, motor neuron-specific transcription factor) and ChAT (choline acetyltransferase) (*Fig. 4 D-I*). Thus, patient-specific iPSC lines are capable of direct differentiation into motor neurons, which are affected during ALS.

Additionally, when using iPSC lines for astroglial differentiation, we identified the cells expressing a specific astrocyte marker (GFAP) (*Fig. 4J*); their toxic effect was displayed in ALS pathology.

Thus, patient-specific iPS lines have an advantage over other published models [25], since these cells are genetically identical to primary patient cells and, potentially, might most accurately reproduce the molecular events taking place in the development of ALS. In addition, cultivation of these iPSC lines under specified conditions (such as the presence of the mTeSR1 medium) allows us to maintain their pluripotent state for a stable and unlimited production of motor neurons.

## CONCLUSIONS

We have obtained iPSC lines from patients with SOD1- associated ALS. The reprogramming efficiency with the lentiviral gene delivery system was at least 10-fold higher than that with the recombinant Sendai virusbased system. These iPSC lines with mutations in the* SOD1 *gene are pluripotent and capable of directed differentiation into motor neurons.

## Abbreviations

ALS: amyotrophic lateral sclerosis
DAPI: 4’,6-diamidino-2-phenylindole
CCS: copper chaperone for superoxide dismutase
ChAT: choline acetyltransferase
ESCs: embryonic stem cells
GFAP: glial fibrillary acidic protein
iPSCs: induced pluripotent stem cells
hSOD1: human superoxide dismutase 1
